# Study protocol for promoting respectful maternity care initiative to assess, measure and design interventions to reduce disrespect and abuse during childbirth in Kenya

**DOI:** 10.1186/1471-2393-13-21

**Published:** 2013-01-24

**Authors:** Charlotte Warren, Rebecca Njuki, Timothy Abuya, Charity Ndwiga, Grace Maingi, Jane Serwanga, Faith Mbehero, Louisa Muteti, Anne Njeru, Joseph Karanja, Joyce Olenja, Lucy Gitonga, Chris Rakuom, Ben Bellows

**Affiliations:** 1 Population Council, P.O Box 17643–00500, Nairobi, Kenya; 2 Federation of Women Lawyers, P.O. BOX 46324–00100, Nairobi, Kenya; 3 National Nurses Association of Kenya, P.O BOX 49422–00100, Nairobi, Kenya; 4 Ministry of Public Health and Sanitation, Division of Reproductive Health, P. O. Box 43319–00100, Nairobi, Kenya; 5 Ministry of Medical Services, Department of Nursing, P.O. Box: 30016–00100, Nairobi, Cathedral Road, Nairobi, Kenya; 6 University of Nairobi, P. O. Box 19676, Nairobi, KNH, Kenya; 7 Kenya Obstetrical and Gynecological Society, P.O Box 19459–00202, Nairobi, Kenya

**Keywords:** Disrespect and abuse, Skilled birth attendant, Childbirth, Implementation research, Kenya

## Abstract

**Background:**

Increases in the proportion of facility-based deliveries have been marginal in many low-income countries in the African region. Preliminary clinical and anthropological evidence suggests that one major factor inhibiting pregnant women from delivering at facility is disrespectful and abusive treatment by health care providers in maternity units. Despite acknowledgement of this behavior by policy makers, program staff, civil society groups and community members, the problem appears to be widespread but prevalence is not well documented. Formative research will be undertaken to test the reliability and validity of a disrespect and abuse (D&A) construct and to then measure the prevalence of disrespect and abuse suffered by clinic clients and the general population.

**Methods/design:**

A quasi-experimental design will be followed with surveys at twelve health facilities in four districts and one large maternity hospital in Nairobi and areas before and after the introduction of disrespect and abuse (D&A) interventions. The design is aimed to control for potential time dependent confounding on observed factors.

**Discussion:**

This study seeks to conduct implementation research aimed at designing, testing, and evaluating an approach to significantly reduce disrespectful and abusive (D&A) care of women during labor and delivery in facilities. Specifically the proposed study aims to: (i) determine the manifestations, types and prevalence of D&A in childbirth (ii) develop and validate tools for assessing D&A (iii) identify and explore the potential drivers of D&A (iv) design, implement, monitor and evaluate the impact of one or more interventions to reduce D&A and (v) document and assess the dynamics of implementing interventions to reduce D&A and generate lessons for replication at scale.

## Background

Pregnancy, childbirth, and their consequences are still the leading causes of death, disease and disability among women of reproductive age in developing countries. Maternal mortality is highest in Sub-Saharan Africa, where the maternal mortality ratio (MMR) is one hundred times greater than in developed regions [1]. One key strategy to address high maternal and newborn morbidity and mortality is to increase the proportion of births attended by skilled birth attendants (SBA); indeed, this is an indicator for United Nations Millennium Development Goal (MDG) 5. Progress has been slow towards achieving this MDG indicator [1] because improvements require overcoming financial and geographic barriers to accessing SBA, as well as poor quality of care at facilities [1,2].

**Figure 1:**
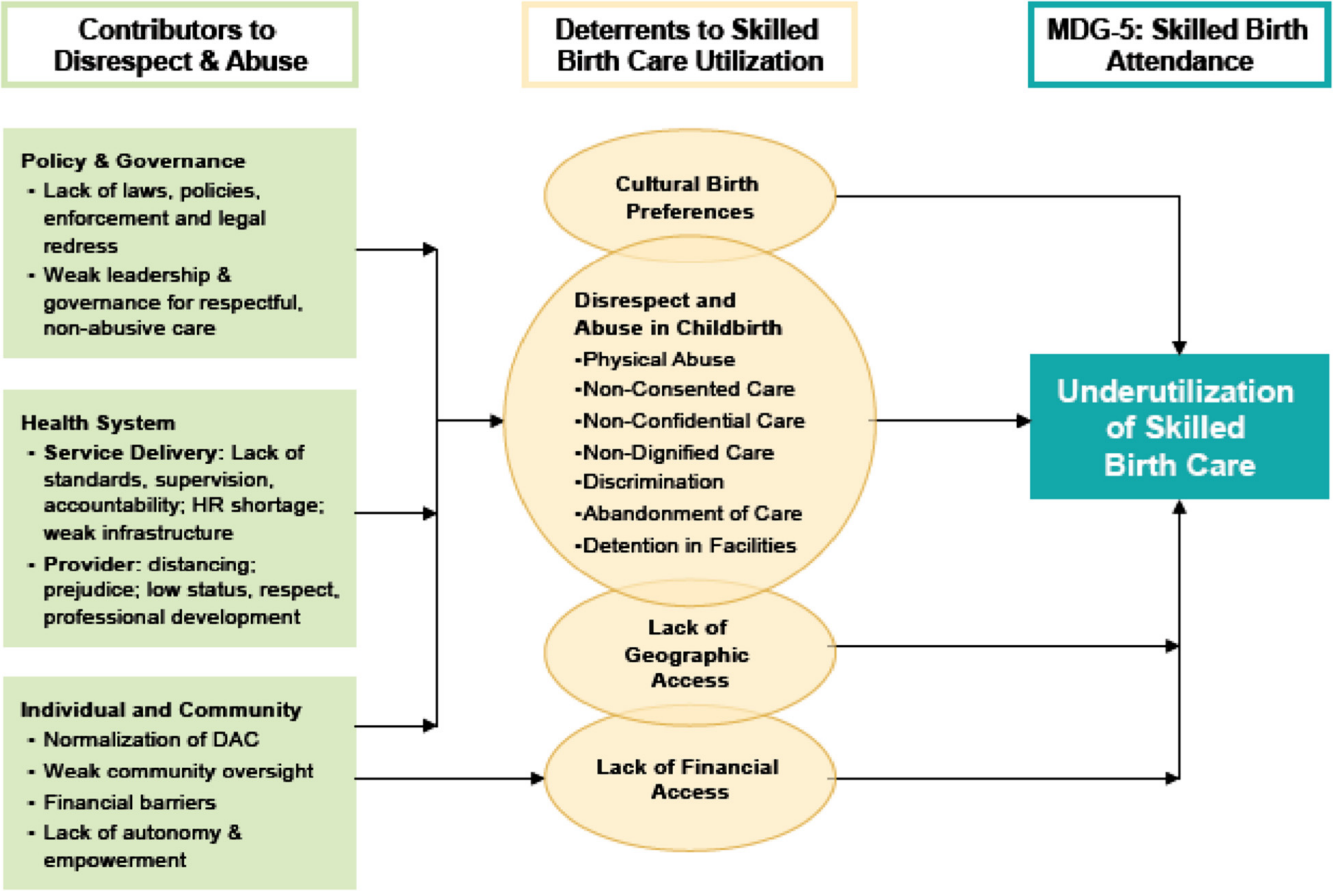
Contributors to and Impact of Disrespect and Abuse in Childbirth (D&C) on Skilled Care Utilization (Bowser and Hill 2010).

An important, but little understood component of the poor quality of care experienced by women during childbirth in facilities is disrespectful and abusive behavior by health workers and other facility staff [3]. Bowser and Hill’s landscape analysis, which explored the evidence of disrespect and abuse during facility based childbirth in 2010, categorized these behaviors into seven types: physical abuse, non-consented care, non-confidential care, non-dignified care, discrimination, abandonment of care and detention in facilities. Numerous factors can contribute to this experience that Bowser and Hill and others group into: individual and community-level factors normalizing D&A, lack of legal and ethical foundations to address D&A, lack of leadership, lack of standards and accountability, and provider prejudice due to training and lack of resources [4] (Figure 1).

Despite acknowledgement of these behaviors by policy makers, program staff, civil society groups and community members, the problem appears to be widespread but prevalence is not well documented. Given the variety of forms it can take and the multiple contributing factors, coupled with a lack of research describing the practice and evaluating interventions, little is known about effective and appropriate interventions that can reduce D&A. Bowser and Hill give some examples of interventions that have been tested including: facility and system-wide quality-improvement initiatives; accountability mechanisms at the facility- and community-levels; protocols and training programs for providers; adaptation of childbirth interventions by communities; and rights and legal campaigns at the national-level [5]. Public awareness and debate on this issue is high, with stories in the national press highlighting the problem [6]. This existing evidence and public awareness provides a strong platform for understanding D&A and initiating sustainable interventions.

In 2007 the Kenyan Federation of Women Lawyers (FIDA) with the Centre for Reproductive Rights documented dimensions of D&A during childbirth, including: physical abuse, non-consented care, non-dignified care, discrimination of poor and young, abandonment of women during and after labor and detention in facilities because of inability to pay including women with stillbirths [7]. In another study in Kenya, Family Care International found that women did not attend facilities for fear of being insulted, roughed up and/or abandoned [8]. Population Council research has also found that negative attitudes of nursing staff and absence of medical personnel at facilities act as barriers to women receiving timely care and attention from SBA [9]. Another recent study in Kenya found that D&A of women during and immediately after childbirth discourages women from attending facility-based births and reported that traditional birth attendants (TBAs) accord them more respect [10]. This was reiterated in the focus group discussions with women in the Kenya Service Provisions assessment 2009/2010 [11]. These women are also likely to discourage others against using facility based services for birth. Moreover, the poorest women and those from ethnic minorities who are most in need of this care, and most likely to benefit from improved quality, are those who are treated with the greatest disrespect. This implementation research aims at designing, testing, and evaluating an approach to significantly reduce disrespectful and abusive care of women during labor and delivery in facilities.

### Study design and methods

#### Study aims, objectives and indicators

The overall aim of the proposed project is to conduct implementation research aimed at designing, testing, and evaluating novel approaches with the potential to significantly reduce disrespectful and abusive care of women during labor and delivery in facilities. Specifically, in the project, we aim to:

1. Determine the manifestations, types and prevalence of D&A in childbirth.

2. Develop and validate tools for assessing D&A.

3. Identify and explore the potential drivers of D&A.

4. Design, implement, monitor and evaluate the impact of one or more interventions to reduce D&A.

5. Document and assess the dynamics of implementing interventions to reduce D&A and generate lessons for replication at scale.

#### Study setting

We propose to undertake this project in Kenya where progress towards improving key skilled birth attendance (SBA) indicators has stalled and interventions are urgently needed to accelerate progress if Kenya is to reach its MDG target of 90% SBA and reduction in MMR to 147 per 100,000 live births by 2015 [12,13]. While most of Kenya’s neighbors are reporting some progress in improving proportions of births with SBA (for example in Uganda the proportion increased from 38% in 1995, 42% in 2006 and stagnated at 41% in 2010; Tanzania increase from 36% in 1999 to 43% in 2004/5 and 50% in 2010; and Rwanda increased from 31% in 2000 to 52% in 2008/9) and 69% in 2010, in Kenya the SBA rate has actually reduced from 50% in 1989 to 44% in 2008/9 [14]. This stagnation is a likely contributor to the sustained high MMR in the country (currently 488 per 100,000 live births).

This project builds on previous research regarding the impact of the voucher and accreditation approach on improving reproductive health behaviors and reproductive health status [15] in five developing countries (Kenya, Tanzania, Uganda, Bangladesh and Cambodia). The advantages of linking this present study with the voucher research in Kenya include the following; (i) Recent data from population surveys across the three districts in Kenya implementing the voucher program and three control districts indicate that disrespect and abuse of women during childbirth and immediately after is hindering the program in achieving its goal of increasing the number of facility-based births, compared to voucher control sites the voucher is protective of women from disrespect and abuse [10]. The wealth of information on D&A being gathered through the voucher evaluation provides a solid foundation from which to develop quantitative measures and test interventions to reduce disrespect and abuse.

## Methods

A quasi-experimental design will be followed in which health facility assessments will be undertaken in six health facilities and their catchment areas in three districts and in one large maternity hospital in Nairobi. A before and after design will be undertaken in the intervention area and among an equivalent comparison population living in areas and utilizing health facilities not served by a D&A program in order to control for potential time dependent confounding at least among observed characteristics. The study population will comprise health facilities, health providers and managers in selected facilities, national level managers and policy makers, women in labour and postpartum women and community members. The sites will be divided into those providing standards services, voucher program only sites, voucher and D&A sites and D&A sites only.

### Proposed data collection procedures

All data collection instruments and procedures will be thoroughly pilot-tested prior to being used in settings similar to those in which they will be administered. Guidance will be sought from women who have recently given birth when finalizing the instruments to ensure that questions and the data collection procedures are conducted with appropriate sensitivity and are not perceived as stigmatizing. The study tools will be pre-tested among a small group of women with similar characteristics as the study population to identify potentially negative consequences and modified accordingly. Study tools for clients will be translated and pretested in the relevant local language. Overall the study design will comprise the following methods:

#### Focus groups discussions (FGDs)

Three to five FGD will be conducted with community members in each district. These will include women who had facility-based birth and those who delivered at home, family members, community health committee members, and local women’s and civil rights groups. Potential participants will be recruited from the communities around the health facilities. Standard FGD procedures will be followed; a group moderator will conduct the session, accompanied by a note-taker. The FGDs will explore broad themes around (i) motivations for giving birth in health facility and selection/use of the particular facility (ii) attitudes towards health facility deliveries (iii) quality and satisfaction with communication/interaction with the provider and (iv) perceptions of health system stigma and discrimination towards those accessing maternal health services. In addition the FGDs will explore and clarify views, opinions and perceptions and enhance the findings from client exit surveys and address unforeseen questions arising from other components of the study. The information generated from the FGDs will be used to; (i) focus and refine the nature, manifestations and indicators of D&A generated through the literature / document review and policy analysis; and (ii) to inform the development of baseline indicators that will measure the effect of interventions at end line.

#### In-depth interviews and informal discussions with senior health managers

We will conduct in-depth interviews with 25 senior reproductive health program managers at national and regional level; district health management teams (DHMTs), facility managers and civil society leaders. The objectives of the interviews will be to collect information similar to that collected through FGDs, with additional information on system level and governance factors that may contribute to abuse and disrespect.

#### Health facility assessments

We will conduct health facility assessments which comprise; (i) interviews with providers (ii) review of inpatient records (iii) structured facility inventory (iv) service statistics (v) observations of client-provider interactions during labour and childbirth and (iv) client exit interviews. We will conduct provider interviews to measure their perceptions of working conditions, respect, empathy with and prejudice against clients, and awareness of policy and service delivery guidelines concerning respect, dignity and client rights. Staff turnover, absenteeism, vacancy rates, workload, motivation and the challenges of managing and retaining maternity staff will also be measured through adapting self-administered instruments developed and validated in South Africa [16]. All providers working in the MCH or Maternity units will be interviewed on their knowledge, practice and attitudes. Lickert scales will be used and providers given the opportunity to self administer part of the interview. The first part of the questionnaire will be administered by the researcher, a separate section with a range of statements covering the various issues will be given to the provider and asked to tick responses ranging from strongly agree to strongly disagree.

(i) *Reviews of inpatient records* will be used to assess the response to complications. We will conduct record reviews with maternity inpatient notes/partographs specifically to record for example, time of diagnosis of obstructed labor and time of cesarean section to measure aspects of untimely care and neglect. We will randomly review the delivery register and extract every fifth inpatient notes for review.

(ii) *Structured facility inventory* will be used to measure information on facility infrastructure, staffing levels, skills and training, and availability of equipment, commodities, test kits, data collection tools, as well as protocols for service delivery (guidelines, policies and standards).

(iii) * Service statistics*, and reports of critical care behaviors where available, will be extracted to measure trends in the number of facility-based births and analyzed using time series charts [17].

(iv) * Observation of client provider interaction* during labour and delivery will measure both the process (how clients are treated and whether they actively participate) and the content (what they are told, technical competence, accuracy of information, provision of essential information during consultations). A structured non-participant observation will be undertaken of client-provider interactions for those clients recruited following the client’s consent. A structured checklist will be used in the observation of the client provider interaction to record their observations of the actions taken by the provider. The checklist comprises a set of items that together comprise pre-determined, agreed-upon and validated indicators of quality of care during labour and delivery. We will then observe the labor and normal delivery of all consenting clients per facility attending for childbirth over a two-week period. There is a precedent for doing this in Kenya, as the recently-completed Service Provision Assessment (SPA) observed normal deliveries using tools we adapted for this study [11].

(v) *Exit interviews of postpartum women* leaving the maternity unit: We will conduct exit interviews with all women aged 15–45 years who have recently delivered, at the selected facilities as they leave the maternity units after giving birth. Exit surveys are aimed to measure the prevalence of D&A during childbirth. All women satisfying these inclusion criteria will be recruited until the required sample sizes had been reached.

To estimate the sample size for evaluating the ultimate outcome indicator of the intervention package, “the reduction in the prevalence of D&A in facilities” we will use an estimated prevalence of 0.2 of women not using facilities due to provider related reasons, based on literature and regional experience where an additional study on D&A is also taking place. This is based on results from a voucher evaluation baseline population survey conducted in 2010 across the six districts, indicated that 22% of women said they did not seek medical care because of unfriendly providers. Therefore calculation of the sample size is based on the estimation that 20% of pregnant women who do not use a facility for delivery is due to provider based reasons. To measure a 10% decrease of D&A, with 80% estimated power for one-sample comparison of proportion with two sided alpha of 0.005 we will require a sample size of 381 for the exit survey for each district.

The distribution of the types of abuse will be assessed in terms of key socio-demographic variables such as age, parity, marital status, education, economic status, ethnicity, and other characteristics identified through the qualitative research that may be associated with discrimination or abuse.

#### Case narratives at community level

We will document examples from women who have experienced D&A and others who have not experienced D&A during childbirth. Women will first be interviewed exiting the postnatal ward and recruited for a follow-up interview at their home or a mutually convenient location within four weeks of childbirth to ask questions regarding their experience (using note taking and tape-recording).

### Validation of data collection tools

We will develop a standard client exit questionnaire to assess abuse and disrespect in facilities that will be validated. This will aim to permit the instrument to be used in similar settings to assess prevalence and types of abuse and disrespect. To validate this tool a series of activities will be conducted. The process will constitute the following steps:

1. Finalizing a Construct Map (matrix) aimed at identifying the measureable elements of disrespect and abuse (Table 1).

**Table 1:** Construct map (matrix)

**Type of abuse**	**Legal definition (where it exists)**	**Observable element**	**Examples**
Physical abuse	The right not to be subjected to cruel, inhuman, or degrading treatment		Pinching /Slapping/Pushing/Beating
			Stitching episiotomy without anesthesia
			FGM during labor/Re-stitching FGM scar
			Rape/ Inappropriate touching during exam- genital/thighs
Non consented care	Medical procedures that are performed without a client’s consent may constitute an actionable tort of “trespass” to the patient’s body.	A woman’s right to information is respected	Non explanation of medical procedures e. g Tubal Ligation, hysterectomy
			“Staff take time to explain: procedures, diagnosis, progress, results, options”
			“Information is given in an open and friendly manner”
			“Clients are encouraged to ask questions”
Non dignified care	The right to dignity: “Every individual shall have the right to the respect of the dignity inherent in a human being”.	A woman’s right to dignity is respected	Use of non dignified language/not addressed rudely “staff are polite and use appropriate language”
			Threats e.g. if you do not cooperate I take you to theater
			Failure to provide services due to personal values
		A woman’s right to information is respected	No explanation of the scope of services offered
			No choice of gender of provider,
			Not exposed unnecessarily
			Un hygienic conditions: Bed sharing/No change of linen/Several babies sharing incubators/Mothers being asked to clean delivery couches/Dirty bathroom/toilets
Discrimination	The right to be free from discrimination		Mothers record clearly marked HIV positive
			Failure to provide medical procedures to HIV clients e.g. limit VE exam done
	The rights to equality and non-discrimination		Denial of services due to lack of money, poverty
Abandonment /neglect	The right to health	“Every woman has access to skilled attendance during delivery”	Delay in receiving care after a decision has been made e.g. to perform C/S
	The Penal Code provides that any person who renders medical or surgical treatment “in a manner so rash or negligent as to endanger human life or to be likely to cause harm to any other person” is guilty of an offence		Failure to provide supplies even if the supplies are available
			Failure to offer service even when the staffs are adequate on duty
			Failure to examine clients/mothers according to the national guidelines even when the resources are available
			Neglect post delivery
Detention	The right to liberty and		When a woman is unable to pay if the baby is sick- welfare of the mother in the facility
	security of person The right not to be detained for non -payment of debt		“Payment for health care services, as well as services related to the underlying determinants of health, has to be based on the principle of equity, ensuring that these services, whether privately or publicly provided, are affordable for all, including socially disadvantaged groups.
Non confidential	The right to privacy and family	A woman’s right to privacy and confidentiality is respected	“ history taking and examination is done in as much privacy as possible”
			“Staff actively protect women’s privacy /confidentiality”
			“Every woman is examined or attended to behind screens”
			“Staff do not discuss or disclose client information to non-health care staff”

2. Conducting a series of interviews with clients to identify potential gaps in the Construct Map. This will be established by inviting 20 women from the focus groups and other women in the community identified by focus group participants who had experienced abuse during facility childbirth in the past year and 20 women with facility delivery in the past year who had not experienced abuse to complete a structured questionnaire. These women will then participate in a semi-structured in-depth interview by a different interviewer, who will be blinded to the responses of the questionnaire. The experiences of specific types of abuse established in the semi-structured interview will be used as the gold standard. We will then compare the results of the in-depth interviews with the results of the exit questionnaire to calculate the sensitivity, specificity, and the positive predictive value of the questionnaire for identifying any abuse and specific categories of abuse.

3. Finalizing the prototype exit interview questionnaire based on the pretested tool and the focus group discussions. This will help in clarifying any gaps in the client exit questionnaire.

4. Assessing the reliability of the exit interview. The questionnaire for “disrespect and abuse” is required to be consistent over the intended survey population. Reliability testing will be conducted to reduce the measurement error. These methods outlined will be used to reduce error caused by influences associated within respondents and the structure of the instrument.

5. Assessing the validity of the questionnaire. We will gather evidence for validity in three ways through internal structure, items analysis, and external measures. We will collect validity evidence based on internal structure evaluating whether the observed data (responses) are consistent with expectations both qualitatively and empirically using rank order correlation. We hypothesize that the items will arrange themselves from low to high along the D&A scale. We will then conduct item analysis (item by item) to determine whether the locations of the respondents for one item performed as expected on other items, comparing mean locations. This is a test of items design. We hypothesize that the mean location of each group will tend to increase as the scores increase.

### The intervention

The consortium for “Measuring D&A in Childbirth in Kenya” will be comprised of Population Council (PC), Federation of Women Lawyers – Kenya (FIDA) and the National Nurses Association of Kenya/Midwifery Chapter (NNAK/MC). This team was strategically created to bring complementary expertise in promoting women’s rights through local and national advocacy (FIDA), empowering health service providers to provide quality care (NNAK/MC), and implementation research to document and learn from the process (PC). Other critical stakeholders include the Ministry of Medical Services, Ministry of Public Health and Sanitation, the White Ribbon Alliance–Kenya and the Health Rights Advocacy Forum (HERAF).

Our strategy for maximizing the likelihood that successful interventions can be institutionalized is to address the five categories of contributing factors identified by Bowser and Hill through engaging with stakeholders at three levels: policy and governance, health system, and community. The landscape analysis that was conducted by Bowser and Hill of D&A in facility-based childbirth details how these levels are interrelated and why they need to be addressed simultaneously [5].

Findings from the baseline study will inform development of the intervention package so that the most prevalent categories of D&A in Kenya are addressed directly. The design will be guided by a human rights-based approach, in particular, by analyzing and addressing the inequalities, discriminatory practices and unjust power relations between providers and clients as defined by international human rights treaties and corresponding governmental obligations and laws. In addition, the existing Kenya quality model and the national maternal care standards will be used to guide the interventions relevant at each level care of service delivery. We will conduct community dialogue meetings to share and validate the findings. In addition we will invite key stakeholders from all levels to a meeting at which key findings will be shared and discussed, contributing factors identified and prioritized. Broad approaches for the intervention package at national, facility and community levels will be discussed and agreed upon by the stakeholder group. To support this process, issues emerging from the data will be organized into the five broad categories of contributing factors at the three levels: Policy and Governance, Health System and Community, wherever possible linking these factors directly with the types of D&A experienced in the Kenyan context (Table 2).

**Table 2:** Proposed three-level intervention

**Intervention level**	**Contributing factors**	**Stakeholders**
Policy and Governance	National laws and policies, Human rights and ethics guidelines	FIDA, PC, Ministries of Health, Gender, HERAF, Women’s groups
	Governance and leadership	Ministries of Health, NNAK/MC, Population Council
Health System	Service delivery structures and sites	Ministries of Health, KMTC, NNAK/MC/MC, KOGS, KNC, PC
	Provider practices and attitudes	MOH, NNAK/MC/MC, KOGS, Population Council
Community	Individual and community attitudes and behaviors	FIDA, HERAF, CBOs/FBOs, women’s groups, gate keepers

### Data management and analysis

Paper questionnaires and PDAs will be used to capture quantitative data. Checklists for the facility inventory and observations will use paper questionnaires whereas the client exit interview will be carried out using PDAs. Data from paper questionnaires will be keyed into Epidata 3.1 and exported into Stata 10 for analysis. Data from PDAs will be downloaded into an MS Access database before being exported into Stata 10 for analysis. Tests of proportions and relationships (between control and experimental or pre-intervention and post-intervention periods) will be made at 1% and 5% level of significance. We will use statistical analyses such as logistic regression models (with any form of D&A as dependent variable) and Z-tests examining D&A rates before and after to test the hypothesis described above, as well as to evaluate the impact of the intervention package on indicators measuring the key contributing factors. Additionally, multi-level regression models will evaluate which individual and group level factors are associated with a woman’s experience of D&A in childbirth, with the primary explanatory variable being which catchment area woman lived in.

Qualitative data will be captured on paper and audio tapes and later transcribed, translated and typed into MS Word, before being exported into QSR NVivo 10 software management and analysis. A thematic framework will be used in qualitative analysis, allowing for iterative use of both deductive and inductive approaches. We will compare analysis charts within and across sites to look for similarities and differences to support identification of key issues around abuse and disrespect. Final qualitative analysis will be organized around a description of the nature, manifestations and experiences at baseline and factors contributing to the abuse.

### Ethical issues

All researchers and research assistants will be trained on the conduct of ethical procedures and will be monitored during field work by Population Council.

Informed consent will be obtained separately for each study participant for each component. All participants will be given detailed information about the study including: aims/methods of study; institutional affiliations of the research; anticipated benefits, risks/discomfort it may cause (expected to be minimal) and follow-up of the study; the time the questionnaire or interview will take; the fact that they may choose not to answer any questions and that they have the right to abstain from participating in the study, or to withdraw from it at any time, without reprisal; measures that will be taken to ensure confidentiality and anonymity of information provided; the conduct of interviews in places of the participant’s choosing and which maximize audio privacy; contact details of the study coordinator for any questions or concerns.

All data will be stored in password protected computer files. Hard copies of questionnaires, anonymised transcriptions and tapes of the group discussions will be stored securely in a locked cabinet, in accordance with the Population Council policy and the Kenya Data Protection Policy.

### Ethical clearance

The research protocol has been reviewed by key stakeholders and ethical clearance has been granted by the Kenya Medical Research Institute (KEMRI) Ethical Review Board (approval number SCC No 288), the Population Council’s Institutional Review Board (No.517), and the Division of Reproductive Health, Ministry of Public Health and Sanitation and the Ministry of Medical Services.

## Discussion

It’s often argued that hospital settings are organized to provide the safety for mothers and babies during childbirth, but this is not always true in Kenya where many facilities are unable to provide minimum requirements for safe delivery, limited staff and a community with little knowledge of their health rights. Respecting a woman as an important and valuable human being and making certain that the woman’s experience during childbirth is satisfying and empowering is a critical process often referred to as “humanizing childbirth”. Humanized birth means the woman is placed in the centre of decision making and providing her information regarding the process and what is happening during childbirth. Dehumanization of childbirth has been experienced and reported in several other countries as a key deterrent to utilization of skilled birth attendance with different manifestation of disrespect and abuse [18].

D&A is a global problem in many low and high income countries although not well documented. Pregnant women seeking maternity care may receive ill treatment that ranges from disrespect of their autonomy and dignity to utter abuse: physical assault, verbal insults, discrimination, abandonment, or detention in facilities for failure to pay. There have been many anecdotal reports but little formative research coupled with a “veil of silence” that has covered up the humiliation and abuse suffered by women seeking maternity care. International human rights do not directly tackle disrespect and abuse as a violation of women’s basic human rights. The White Ribbon Alliance for Safe Motherhood is bringing together concerned partners to develop collaborative strategies to address disrespect and abuse during maternity care.

The Bowser and Hill landscape report reviews a number of studies from a wide range of countries on respectful care and identifies D&A evidence gaps as the lack of: operational definitions; validated measurement methods; evidence of successful interventions; and prevalence estimates [5]. There is a lack of systematic evaluation and analysis of the contributors of D&A and specific mechanisms by which different drivers may contribute to the problem including interactions between the different drivers. Another gap is the specific way in which D&A acts as a deterrent to skilled care utilization as well as the contribution of the different categories of D&A in reducing maternal health coverage. There are almost no studies that evaluate impact of interventions designed to reduce D&A or promote respectful care. One study in high resource setting attempted to measure the prevalence but did not focus on childbirth [19]. This proposed study aims to better understand the extent of the problem and to document effective approaches to designing and implementing interventions to reduce the disrespect and abuse.

## Abbreviations

D&A: Disrespect and Abuse; FGD: Focus Group Discussion; FIDA: Federation of Women Lawyers – Kenya; HFA: Health Facility Assessment; KEMRI: Kenya Medical Research Institute; MMR: Maternal maternity Ratio; NNAK/MC: National Nurses Association of Kenya/Midwifery Chapter; OBA: Output based Aid; PC: Population Council; TBA: Traditional birth attendant.

## Competing interests

The authors declare that they have no competing interests.

## Authors’ contribution

CW is the PI and was involved in the overall conceptual design and overall revision of the manuscript. RN was involved in the conceptual design of the study, drafting, re-organization and overall revision of the manuscript. TA, CN, JS, FM, LM, AN, JK, GM, JO, LG, CR and BB were involved in the conceptual design of the study and revision of the manuscript. All authors have read and approved the final manuscript.

## Pre-publication history

The pre-publication history for this paper can be accessed here:

http://www.biomedcentral.com/1471-2393/13/21/prepub
